# Books and General Orders

**Published:** 1885-12

**Authors:** 


JOURNAL OF HEALTH PUBLISHING CO.,

E. W. CAPRON, Manager.

### BOOKS AND GENERAL ORDERS.

We have perfected arrangements by which we can furnish books of all kinds at publishers' prices. Send us your orders and the postage for the books, if you know the price, or to enquire the price of any book you may wish and we will send you a reply.

We will also purchase at the lowest cash price, articles of *household furniture* from a stove in the kitchen to the finest work of art, at a rate which will be much lower than they can usually be purchased for by the individuals. In household furniture and ornaments, in brass work of all kinds, from the finest ornaments in the market to the stately and substantial bedstead, we can furnish an unlimited choice, and of the finest quality.

Address,

